# Reading-induced shifts of perceptual speech representations in auditory cortex

**DOI:** 10.1038/s41598-017-05356-3

**Published:** 2017-07-11

**Authors:** Milene Bonte, Joao M. Correia, Mirjam Keetels, Jean Vroomen, Elia Formisano

**Affiliations:** 1 Department of Cognitive Neuroscience, Faculty of Psychology and Neuroscience, Maastricht University, Maastricht, The Netherlands; 2 Maastricht Brain Imaging Center, Maastricht University, Maastricht, The Netherlands; 3 Department of Cognitive Neuropsychology, Tilburg University, Tilburg, The Netherlands; 4 Maastricht Center for Systems Biology (MaCSBio), Maastricht University, Maastricht, The Netherlands

## Abstract

Learning to read requires the formation of efficient neural associations between written and spoken language. Whether these associations influence the auditory cortical representation of speech remains unknown. Here we address this question by combining multivariate functional MRI analysis and a newly-developed ‘text-based recalibration’ paradigm. In this paradigm, the pairing of visual text and ambiguous speech sounds shifts (i.e. recalibrates) the perceptual interpretation of the ambiguous sounds in subsequent auditory-only trials. We show that it is possible to retrieve the text-induced perceptual interpretation from fMRI activity patterns in the posterior superior temporal cortex. Furthermore, this auditory cortical region showed significant functional connectivity with the inferior parietal lobe (IPL) during the pairing of text with ambiguous speech. Our findings indicate that reading-related audiovisual mappings can adjust the auditory cortical representation of speech in typically reading adults. Additionally, they suggest the involvement of the IPL in audiovisual and/or higher-order perceptual processes leading to this adjustment. When applied in typical and dyslexic readers of different ages, our text-based recalibration paradigm may reveal relevant aspects of perceptual learning and plasticity during successful and failing reading development.

## Introduction

The acquisition of reading requires explicit instruction and years of practice and is accompanied by a gradual re-shaping of existing brain networks for visual perception and spoken language^1, 2^. During this brain reorganization, higher-order visual regions in the (left) ventral occipito-temporal cortex become increasingly specialized in visual text perception^3–5^. Moreover, superior temporal, inferior parietal and frontal networks mediating spoken language functions become closely linked to these visual regions, building new cross-modal associations^6–9^. Accordingly, it has been suggested that the brain’s reading network is shaped around the establishment of robust and automatic neural mappings of visual symbols (letters, words) onto corresponding spoken language representations (phonemes, words)^10–12^. The present study investigates a possible mechanism of auditory cortical plasticity that may be pivotal to the formation of these mappings.

Previous functional MRI studies indicate that, in fluent readers, posterior superior temporal cortical responses to speech sounds are enhanced by the simultaneous presentation of matching visual letters in comparison to non-matching letters^13, 14^. Furthermore, the amplitude of unimodal (speech) and crossmodal (text-speech) responses in this region has been found to scale with individual differences in phonological and/or reading skills in typical readers^7, 15, 16^ and pre-readers^17^, and to show an overall reduction in dyslexic readers^14, 18, 19^. However, it remains debated whether and how learning to read changes the representation of speech at the level of the auditory cortex^1, 20^.

**Figure 1:**
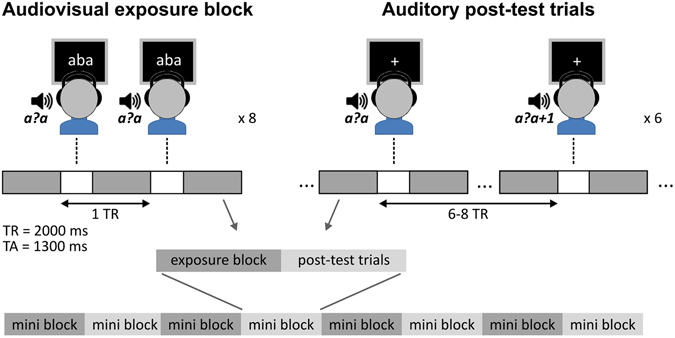
Experimental Design. Schematic overview of the fMRI stimulation protocol and one mini block including an audiovisual exposure block (‘aba’ example) containing 8 presentations of the ambiguous /a?a/ sound together with ‘aba’ text, followed by 6 auditory post-test trials involving the slow event-related presentation of the /a?a/ sound and its two closest neighbours on the continuum. The experiment consisted of 3 runs that each contained 8 of these mini blocks.

Most studies so far have relied on experimental designs involving audiovisual congruency manipulations or higher-order language tasks and used univariate fMRI analyses schemes. Here we employ a newly-developed ‘text-based recalibration’ paradigm in combination with multivariate fMRI decoding techniques, enabling investigating the on-line relation between audiovisual learning and fine-grained auditory cortical representations of speech. Recalibration (or phonetic recalibration) refers to a shift in the perception of ambiguous speech, induced by the prior presentation of visual or other contextual information. Here we use the speech sound /a?a/, where ‘?’ is an ambiguous phoneme midway between /b/ and /d/^21^. When a participant listens to this ambiguous sound, about half of the time he/she perceives the sound as /aba/ and about half of the time as /ada/. In other words, this sound is at the perceptual boundary between /aba/ and /ada/. By pairing the ambiguous sound to disambiguating contextual information one can temporarily shift (recalibrate) this auditory perceptual boundary and bias later perceptions towards either /aba/ or /ada/. So far, most recalibration studies have exploited the naturally evolved audiovisual association between spoken language and lip movements^21, 22^. Other stimuli that have been shown to recalibrate listener’s perceptual speech boundaries include lexical (spoken word) context^23^, and more recently, overt or imagined speech articulation^24^, and written text^25^. During text-based recalibration, repeated pairing of the ambiguous /a?a/ sound to the text ‘aba’ (Fig. 1 – audiovisual exposure block) shifts participants’ later perception of this sound towards /aba/ (Fig. 1 – auditory post-test trials). Likewise, ‘ada’ text shifts later perceptions towards /ada/. Recalibration involves an ‘attracting’ perceptual bias, i.e. the phoneme boundary shifts towards the visual information. In contrast, an opposite ‘repulsive’ perceptual bias (or selective adaptation) is induced after repeated presentation of the same text together with clear speech sounds. That is, after exposure to ‘aba’ text together with clear /aba/ speech sounds, the ambiguous /a?a/ sound is more often perceived as /ada/ (and ‘ada’ text more often leads to /aba/ perception)^25^. Whereas recalibration typically involves the disambiguation of ambiguous speech signals based on short-term perceptual learning, selective adaptation most likely relies on basic auditory sensory mechanisms^22, 26, 27^.

The present study combines psychophysical and fMRI measures of text-based recalibration to investigate text-induced audiovisual plasticity in typically reading adults. First, we study whether text-based recalibration changes the representation of ambiguous speech sounds in the auditory cortex. Because recalibration reflects a shift in *perception* while the acoustics of the ambiguous speech sound remains constant, this requires distinguishing subtle changes in brain activity patterns. FMRI decoding methods ensure high sensitivity to small and spatially distributed effects essential for the detection of this type of subtle changes^28^. A previous study demonstrated that perceptual recalibration of ambiguous speech due to lip-read information could be decoded from fMRI activation patterns in early and higher-order auditory cortex^29^. Here, we use a similar approach consisting of a decoding algorithm to detect changes in brain activity patterns associated with the perception of /aba/ versus /ada/. In addition, we investigate brain regions that may mediate this text-induced recalibration, by performing functional connectivity analysis of brain activity during the audiovisual exposure blocks. Our results show that it is possible to decode - from fMRI activity patterns in the posterior superior temporal cortex - reading induced shifts in /aba/ versus /ada/ perceptual interpretations of the same ambiguous sound. Furthermore, they suggest the involvement of the inferior parietal lobe in audiovisual and/or higher-order perceptual processes leading to this reading-induced auditory bias.

## Results

### Psychophysical experiment

**Figure 2:**
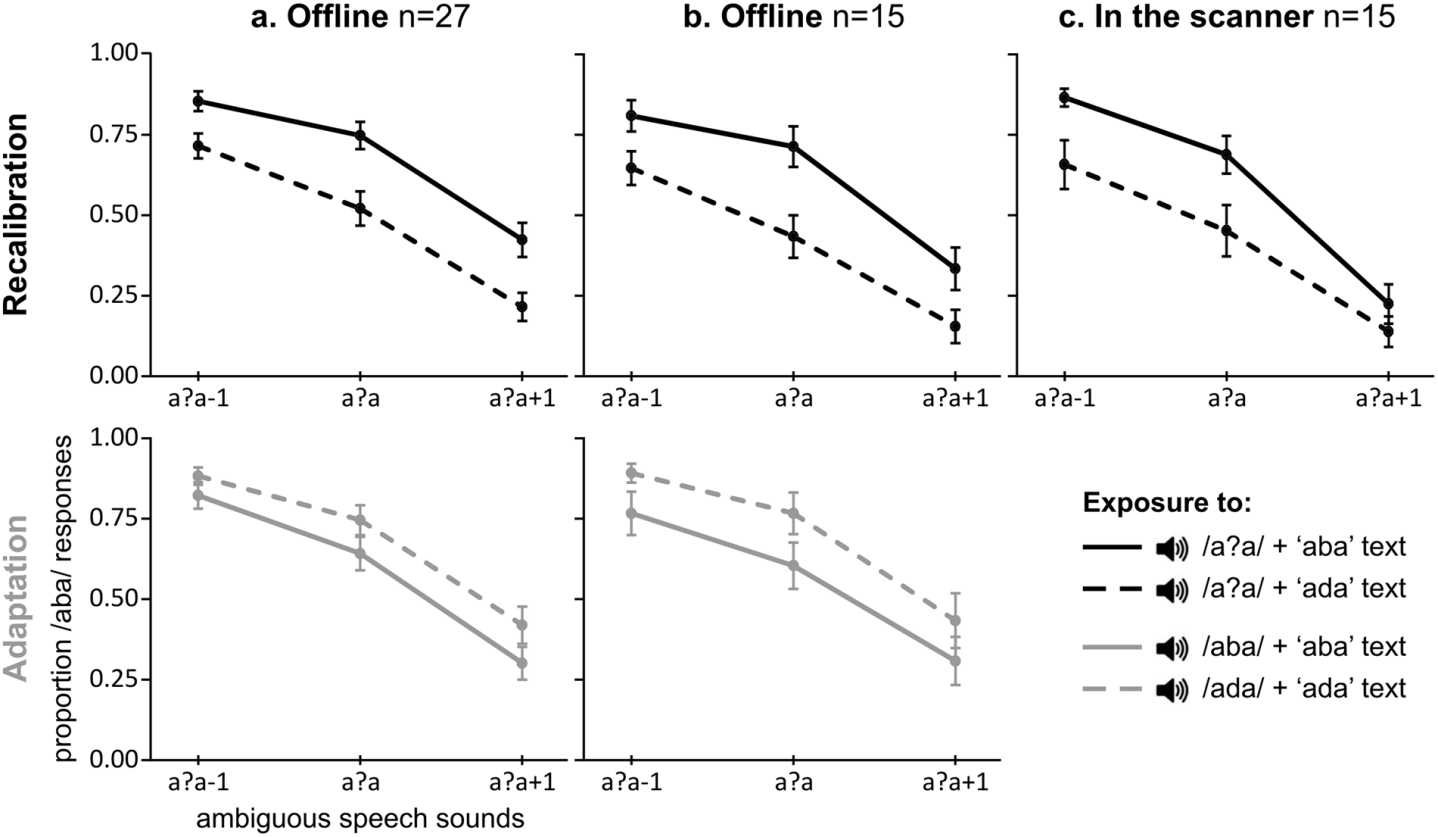
Behavioural results of the auditory post-test trials. The graphs show participant’s mean (standard error) proportion of /aba/ responses for the ambiguous /a?a/ sound and its two closest neighbours on the /aba/-/ada/ continuum (F2 manipulation), following either recalibration (top row) or adaptation (bottom row) exposure blocks. Exposure blocks consisted of repeated pairing of the ambiguous /a?a/ sound (recalibration) or clear /aba/, /ada/ sounds (adaptation) with either ‘aba’ text (solid lines) or ‘ada’ text (dashed lines). During the offline psychophysical experiment participants showed significant and opposite adaptation and recalibration effects (**a**,**b**). The recalibration effect was replicated in the scanner (**c**).

Twenty-seven adult participants performed a psychophysical experiment including both recalibration (Fig. 2, top row) and adaptation exposure blocks (Fig. 2, bottom row). During post-test trials participants listened to the ambiguous /a?a/ sound and its two closest neighbours /a?a/+1 and /a?a/−1 and categorized these sounds as either /aba/ or /ada/ (once the fixation cross turned green, 1 s after sound onset, see Methods). Recalibration exposure blocks (text + ambiguous sound) resulted in an ‘attracting bias’, both in the group of 27 participants and in a subgroup of 15 participants that were included in our subsequent fMRI study (Fig. 2a,b – top row). Exposure to ‘aba’ text (continuous line) shifted participant’s later perception towards /aba/, while ‘ada’ text (dashed line) shifted later perception towards /ada/ (proportion of /aba/ responses of 0.68 vs. 0.48 (n = 27) and 0.62 vs. 0.41 (n = 15), respectively). As expected, adaptation exposure blocks (text + matching clear sound) yielded an opposite ‘repulsive bias’ (Fig. 2a,b – lower row): exposure to ‘aba’ text shifted participant’s later perception towards /ada/, while ‘ada’ text shifted later perception towards /aba/ (proportion of /aba/ responses of 0.59 vs. 0.68 (n = 27) and 0.56 vs. 0.70 (n = 15), respectively).

These recalibration and adaptation effects were confirmed by a statistical analysis based on a generalized linear mixed-effects model with a logistic link function that accounts for our dichotomous dependent variable, i.e. / aba/ vs. /ada/ responses (lme4 package in R version 3.3.3). In this model, /aba/ responses were coded as “1” and /ada/ responses as “0”, therefore positive fitted coefficients correspond to more /aba/ responses (Table 1). Factors were coded to reflect the difference between experimental variables (Condition: recalibration = +0.5, adaptation = −0.5; Exposure: ‘aba’ text = +0.5, ‘ada’ text = −0.5; Sound: /a?a/−1 = +1, /a?a/ = 0, /a?a/+1 = −1). The model included main and interaction effects of Condition, Exposure and Sound as fixed factors (Table 1). Following our recent behavioural text-based recalibration study^25^, model validation was based on the maximal random effect structure supported by the data^30^. Specifically, the random effects structure was tested until model convergence, by starting with a maximal model (random slopes for all main effects and interactions) and removing slopes based on their relevance (i.e. random effect correlations, main effect of Condition, Condition by Sound and Exposure by Sound interactions). The analysis showed a significant Condition by Exposure interaction (Table 1; n = 27: b = 1.78, p = 2e-16; n = 15: b = 2.18, p = 2e-16), confirming the expected opposite perceptual shift following recalibration versus adaptation exposure blocks (Fig. 2ab). The strength of recalibration and adaptation effects did not significantly differ across Sounds (i.e. no Condition by Sound interaction), but results did show an expected overall difference in the proportion of /aba/ responses for the /a?a/−1, /a?a/ and /a?a/+1 sounds (main effect of Sound, n = 27: b = 1.43, p = 2e-16; n = 15: b = 1.41, p = 2e-16). Additionally, in the group of 27 participants, the analysis showed a significant positive effect for the intercept (b = 0.69, p = 0.0009), indicating an overall /aba/ bias, as well as for Exposure (b = 0.26, p = 0.0004), indicating a slight bias towards /aba/ responses after ‘aba’ vs. ‘ada’ text. In our subset of 15 participants, these effects were not replicated, but the analysis instead showed a negative main effect of Condition (b = −0.79, p = 0.0088) indicating an overall /aba/ bias after adaptation vs. recalibration exposure blocks.

**Table 1:** Results psychophysical experiment.

	Fixed Factor	Estimate	SE	z-value	p
N = 27	(Intercept)	0.69	0.20	3.32	0.0009 ***
	Condition (*recalibration*, *adaptation*)	−0.40	0.22	−1.78	0.0750
	Exposure (‘*aba*’, ‘*ada*’ *text*)	0.26	0.07	3.53	0.0004 ***
	Sound (/*a?a*/-*1*, /*a*?*a*/, /*a*?*a*/+*1)*	1.43	0.05	28.81	2e-16 ***
	Condition by Exposure	1.78	0.20	8.92	2e-16 ***
	Condition by Sound	−0.17	0.10	−1.75	0.0804
	Exposure by Sound	−0.01	0.10	−0.09	0.9271
	Condition by Exposure by Sound	−0.14	0.19	−0.73	0.4613
N = 15	(Intercept)	0.50	0.30	1.70	0.0895
	Condition *(recalibration, adaptation)*	−0.79	0.30	−2.62	0.0088 *
	Exposure (‘*aba*’, ‘*ada*’ *text*)	0.12	0.10	1.19	0.2340
	Sound (/*a*?*a*/-*1*, /*a*?*a*/, /*a*?*a*/+*1*)	1.41	0.07	21.20	2e-16 ***
	Condition by Exposure	2.18	0.20	10.70	2e-16 ***
	Condition by Sound	−0.14	0.13	−1.08	0.2800
	Exposure by Sound	−0.14	0.12	−1.12	0.2646
	Condition by Exposure by Sound	0.18	0.25	0.73	0.4683

Fitted model: Response ~1 + Condition * Exposure * Sound + (1 + Condition + Condition:Exposure || Subject). Fixed effects correlations were below 0.18 (n = 27) or below 0.23 (n = 15).

SE = standard error; ***p < 0.001; **p < 0.01; *p < 0.05.

### Behavioural results in the scanner

Behavioural results obtained while 15 participants performed the same task in the scanner, successfully replicated the text-based recalibration effect (Fig. 2c – upper row), leading to a proportion of /aba/ responses of 0.59 after ‘aba’ exposure and of 0.42 after ‘ada’ exposure. The recalibration effect was confirmed by a generalized linear mixed-effects model including main and interaction effects of Exposure and Sound as fixed factors, and main effects of Exposure and Sound as random slopes (Table 2). The analysis showed the expected significant main effect of Exposure (b = 1.14, p = 1.29e-05) as well as a main effect of Sound (b = 1.86, p = 2e-16). Furthermore, a tendency towards a smaller recalibration effect for the /a?a/+1 sound as compared to the other sounds (Fig. 2c – upper row) led to an almost significant Exposure by Sound interaction (b = 0.38, p = 0.0545).

**Table 2:** Behavioural results in the scanner.

	Fixed Factor	Estimate	SE	z-value	p
N = 15	(Intercept)	0.01	0.31	0.03	0.9742
	Exposure (‘*aba*’, ‘*ada*’ *text*)	1.14	0.26	4.36	1.29e-05 ***
	Sound (/*a*?*a*/−*1*, /*a*?*a*/, /*a*?*a*/+*1*)	1.86	0.22	8.45	2e-16 ***
	Exposure by Sound	0.38	0.20	1.92	0.0545

Fitted model: Response ~1 + Exposure * Sound + (1 + Exposure + Sound || Subject). Fixed effects correlations were below 0.06. SE = standard error ***p < 0.001; **p < 0.01; *p < 0.05.

### fMRI activity during the auditory post-test trials

**Figure 3:**
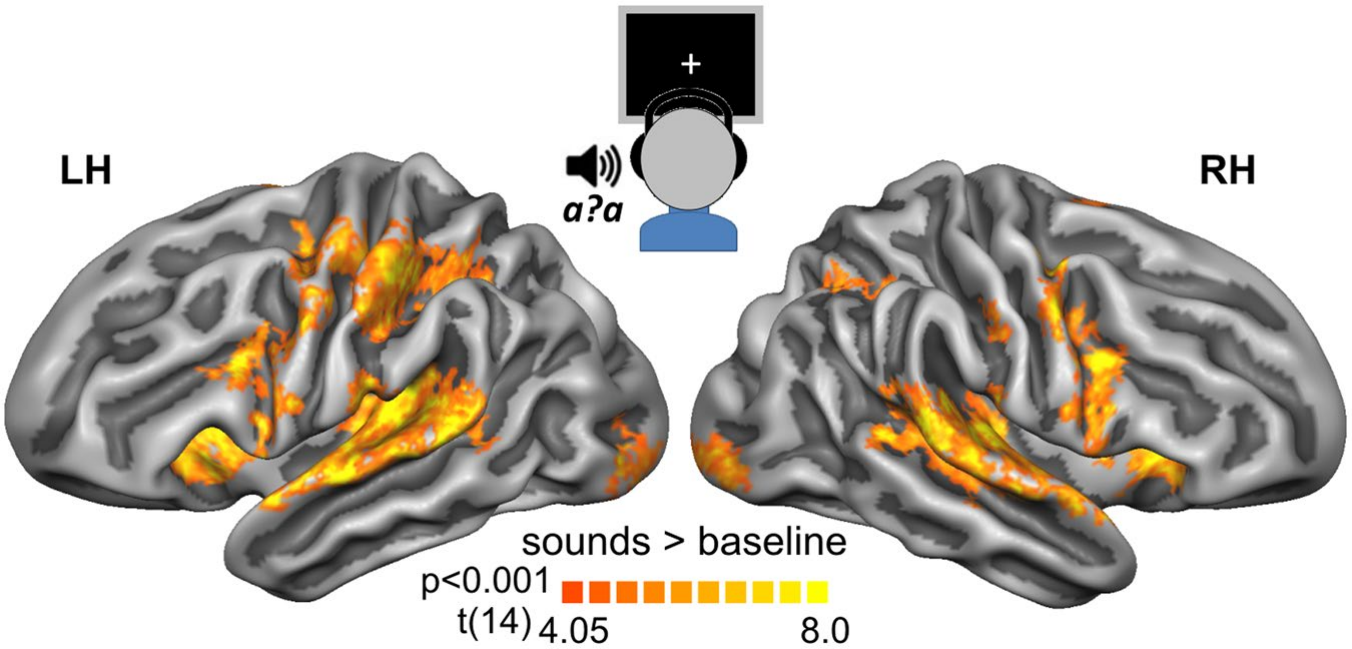
fMRI activity during auditory post-test trials. Functional maps illustrating activity evoked by the ambiguous post-test speech sounds. The maps are based on random effects contrasts, corrected for multiple comparisons using cluster size correction (p_corr_ < 0.05) with a primary threshold of p_vertex_ = 0.001, and visualized on cortical surface representations of the left (LH) and right (RH) hemispheres (light grey: gyri and dark grey: sulci), resulting from the realignment of the cortices of our 15 participants.

During the post-test trials blood-oxygen-level dependent (BOLD) activity was elicited across a broad network of perceptual, motor and fronto-parietal regions, reflecting listening to the ambiguous sounds and making /aba/–/ada/ judgments (once the fixation cross turned green). This network included early (Heschl’s Gyrus/ Heschl’s Sulcus) and higher-order auditory regions as well as primary and extrastriate visual regions (Fig. 3). Superior temporal gyrus (STG) activity extended towards the middle to posterior superior temporal sulcus (STS) and middle temporal gyrus (MTG) especially in the right hemisphere. Other activated regions included somatosensory, motor and premotor areas with more widespread activity in the left hemisphere, as well as the bilateral inferior parietal lobe (IPL), and frontal regions including the insula, inferior frontal gyrus (IFG) and inferior frontal sulcus (IFS). A GLM analysis with trial labelling according to participant’s perception of the ambiguous post-test sounds did not yield any significant univariate activity differences between /aba/ versus /ada/ perceptions.

### fMRI decoding of auditory post-test trials

**Figure 4:**
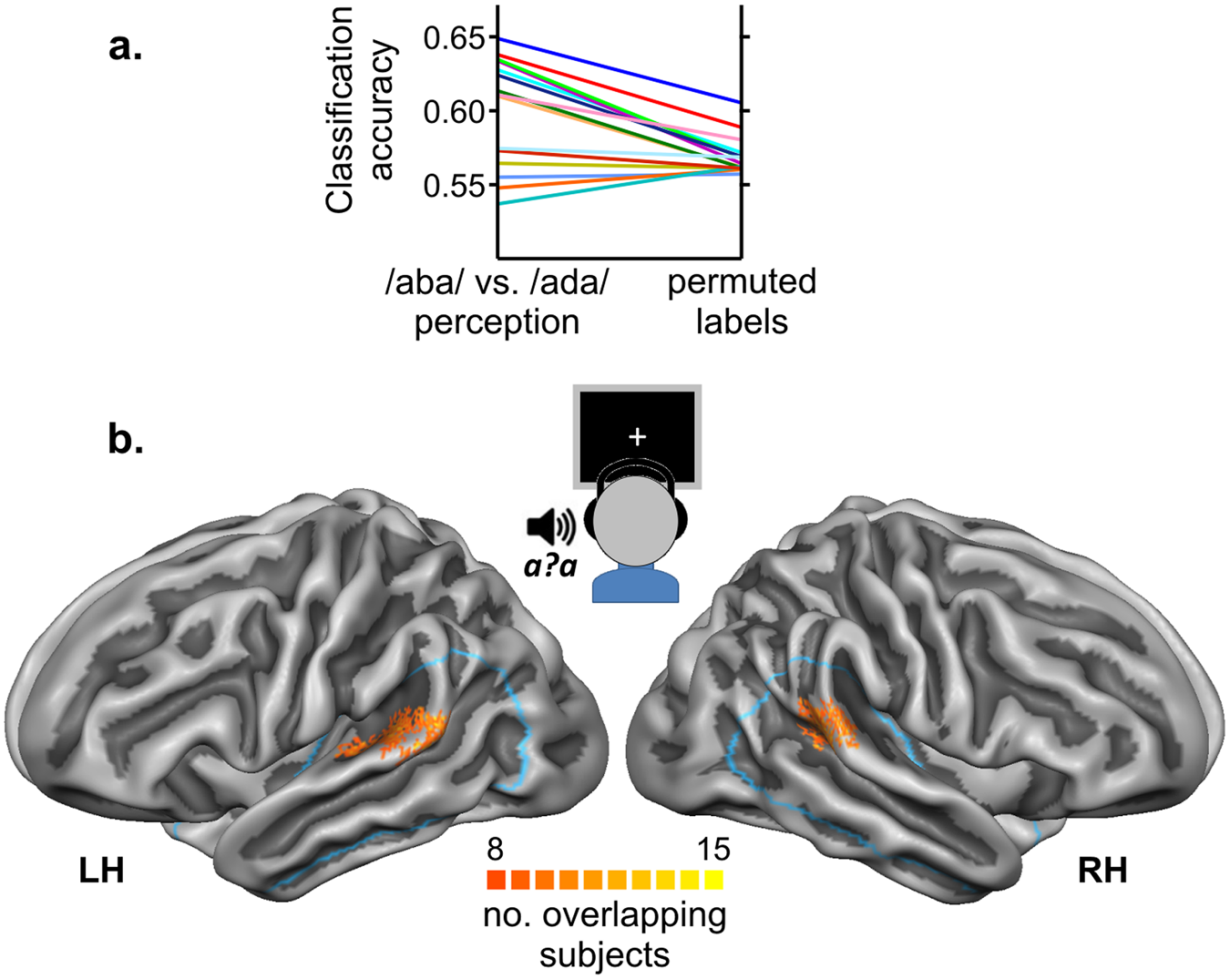
fMRI decoding of the auditory post-test trials. (**a**) Classification accuracies for each of our 15 individual participants. Accuracies obtained with the perceptual labels (/aba/ vs. /ada/ perception) were significantly higher (p = 0.0043) than the accuracies based on permuted labels following the exact same decoding procedure. (**b**) Discriminative maps that illustrate for how many subjects vertices were among the 20% most discriminative features for decoding /aba/ vs. /ada/ perception. Blue outlines delineate the anatomical temporal cortex masks (ROIs) within which fMRI decoding was performed. Maps are visualized on the aligned group-averaged cortical surface representations of the left (LH) and right (RH) hemispheres.

To investigate whether the perceived identity of the ambiguous post-test sounds was reflected in more fine-grained activity patterns, we applied multivariate fMRI decoding techniques (see Methods) within an anatomical superior temporal cortex (STC) ROI (Fig. 4b – blue outline). This analysis showed that it was possible to significantly distinguish STC activity patterns associated with /aba/ vs. /ada/ perceptions (group average accuracy = 0.60; p = 0.0043 with respect to permutation-based chance level). Classification accuracies in individual participants (Fig. 4a) revealed higher than label-permuted accuracies in the majority of participants. Analysis of cortical locations that most consistently contributed to the decoding of perceptual labels across participants yielded a left STG cluster covering part of the planum temporale towards HG as well as a cluster in the right posterior STG/STS (Fig. 4b).

### fMRI functional connectivity during audiovisual exposure

**Figure 5:**
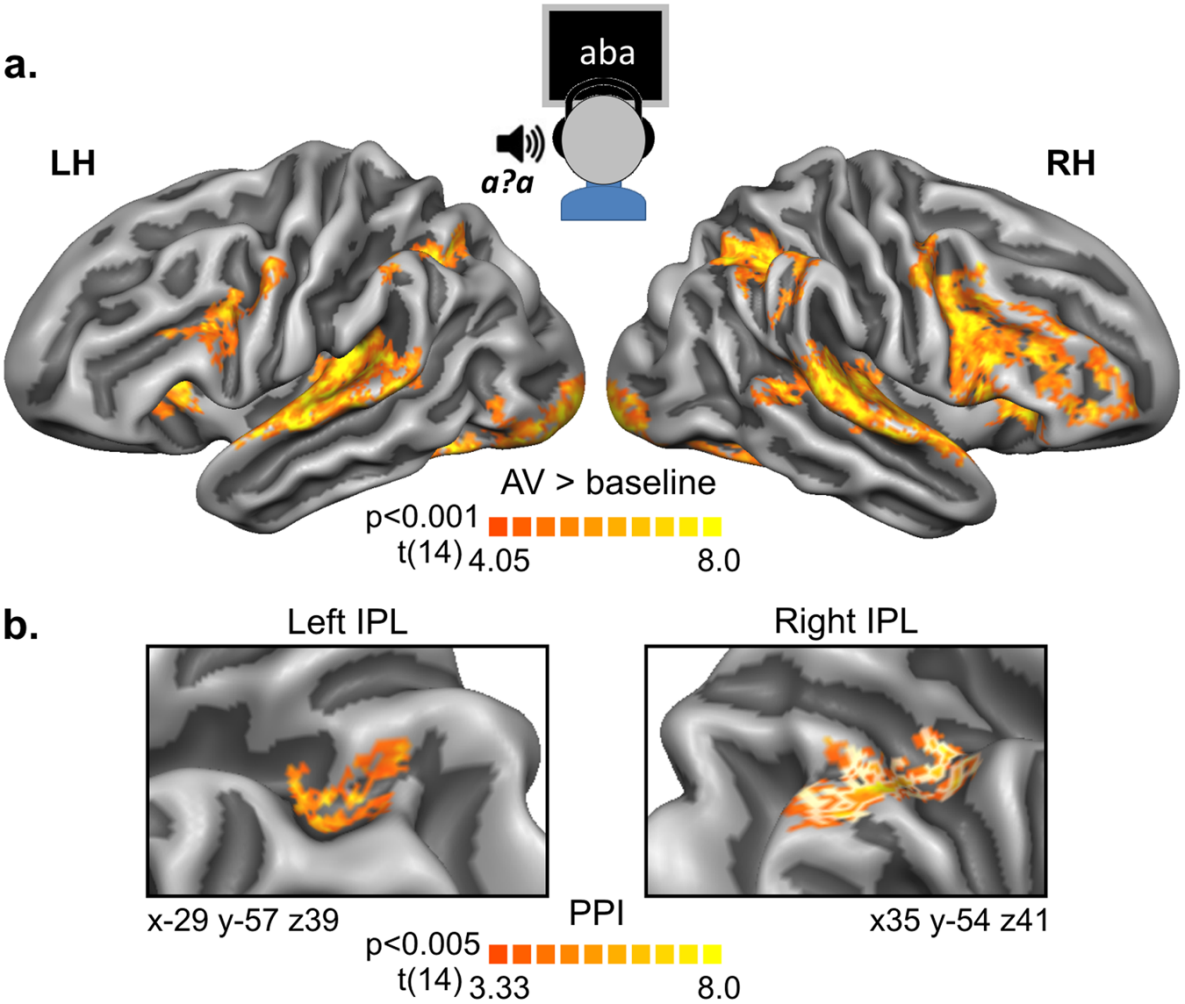
fMRI activity and connectivity during audiovisual exposure blocks. (**a**) Functional contrast maps illustrating overall BOLD responses during the audiovisual (AV) exposure blocks, corrected for multiple comparisons using cluster size correction (p_corr_ < 0.05) with a primary threshold of p_vertex_ = 0.001. (**b**) Psychophysiological interaction (PPI) maps during the AV exposure blocks showing significant clusters in the left and right inferior parietal lobe (IPL) with normalized areas of 58 and 181 mm^2^ respectively. Talairach coordinates (xyz) refer to the centre of gravity of the IPL regions. Maps are corrected for multiple comparisons using cluster size correction (p_corr_ < 0.05) with a primary threshold of p_vertex_ = 0.005. At a primary threshold of p_vertex_ = 0.001 only the right IPL cluster survives (white outlines). All maps are based on random effects contrasts and visualized on group-averaged and aligned cortical surface representations of the left (LH) and right (RH) hemispheres.

During the exposure blocks, paired text and ambiguous speech sound stimuli evoked significant BOLD responses across a network of auditory, visual and fronto-parietal cortical regions (Fig. 5a). To examine the relation between the activity in these regions and the superior temporal regions informative of the auditory perceptual shift, we performed a psychophysiological interaction (PPI) analysis using the left STG cluster (Fig. 4b) as seed region (see Methods refs 31 and 32). The resulting PPI group map (Fig. 5b) indicated significant clusters in bilateral IPL (p_corr_ < 0.05, with a primary vertex-level threshold of p_vertex_ = 0.005), of which the right hemisphere cluster also survived multiple comparisons correction using a primary vertex-level threshold of p_vertex_ = 0.001. These maps thus suggest a rather focal increase in correlation between activity time-courses of the IPL and the posterior STG during audiovisual exposure blocks relative to baseline. At a more lenient threshold of p_vertex_ = 0.05, the PPI group map additionally included clusters in the left inferior occipital gyrus, the right lateral occipitotemporal gyrus, the right precuneus and the right IFG, extending towards the middle frontal gyrus.

## Discussion

We investigated reading-induced audiovisual plasticity by using written text to recalibrate participant’s perception of ambiguous speech sounds. Text-based recalibration resulted in perceptual shifts and subtle changes in auditory cortical activity patterns that were detected by our fMRI decoding algorithm. Functional connectivity analysis of preceding audiovisual activation indicated the involvement of the inferior parietal lobe (IPL) in audiovisual and/or higher-order perceptual processes leading to these text-induced changes. Together, our behavioural and fMRI findings suggest a central role of the auditory cortex in representing reading-related audiovisual mappings.

Our offline psychophysical experiment showed the expected and opposite ‘attracting’ versus ‘repulsive’ bias in the recalibration (text + ambiguous speech) versus adaptation (text + matching clear speech) contexts^25^. The recalibration effect indicates that in experienced readers, both written text and lip-read speech may recalibrate the auditory perceptual boundary of ambiguous speech. Furthermore, the opposite behavioural effect observed in the adaptation context suggests the involvement of distinct underlying mechanisms and controls for a simple response bias or ‘prior’ due to e.g. the perception of one particular sound (e.g. /aba/) during the preceding exposure phase^22, 26, 27^. Whereas phonetic recalibration may result from various natural or acquired stimulus mappings, different types of mappings may differ in the strength of the resulting recalibration effects. For example, recalibration effects are typically reported to be stronger for lipread speech, as compared to lexical speech information^33^, mouthing or speech imagery^24^. Similarly, lipread speech has a stronger effect than visual text^25, 34^. Behavioural findings of our group further suggest that the strength of text-based recalibration is modulated by individual differences in reading fluency, i.e. text-based recalibration was found to be significantly stronger in adults with fluent reading skills than in dyslexic readers^34^. The specificity of this finding was emphasized by the fact that these same groups did not differ when lipread speech was used to induce recalibration. Whether the assignment of arbitrary stimulus mappings (e.g. square for /aba/; triangle for /ada/) also leads to adjustments of the perceptual boundary of ambiguous speech input has not yet been tested. If such newly learnt mapping also induced phonetic recalibration, this would provide a relevant additional means to studying individual differences in perceptual language learning.

In line with the hypothesized perceptual nature of the recalibration effect, our fMRI decoding results demonstrated that recalibration was accompanied by subtle changes in auditory cortical activity patterns. Thus, it was possible to consistently predict whether participants perceived the same ambiguous speech sounds as either /aba/ or /ada/ based on activity patterns in the posterior STG, extending along the planum temporale towards early auditory regions (HG/HS) in the left hemisphere and towards the STS in the right hemisphere. These superior temporal regions have been associated with the processing of isolated speech sounds^35, 36^ and with the representation of speech sounds at different levels of abstraction, including representations that are modulated by task demands^37^ and robust to speaker changes^28, 38^. In particular, response patterns in similar auditory regions were previously shown to be informative of the perceptual shifts induced by recalibration through lipread speech^29^. The finding that visual text temporarily changes the representation of ambiguous speech in the posterior STG/STS, extends previous findings showing that this region’s response to spoken phonemes is enhanced by the simultaneous presentation of matching visual letters in comparison to non-matching letters^13, 14, 17, 18^. The additional involvement of early auditory regions (HG/HS) typically assumed to be restricted to the low-level analysis of acoustic features^39, 40^, emphasizes the basic perceptual nature of the text-induced recalibration effects and indicates the importance of early in addition to higher order auditory regions in the perceptual interpretation of phonemes^28, 37, 38, 41^.

The resemblance of our fMRI decoding results to those obtained with lip-read speech^29^, shows that natural and culturally defined audiovisual associations both modulate auditory cortical representations of speech. This is compatible with the notion that reading acquisition is accompanied by a gradual re-shaping of brain networks for speech perception that become closely linked to higher-order visual regions in the ventral occipital cortex^1^. The presently observed shift in auditory cortical activity patterns may reflect a shift in the phonemic category boundary towards either /b/ or /d/, e.g. along the F2 formant that was used to create the /aba/-/ada/ continuum^42^. Whereas the employed fMRI decoding techniques allowed discriminating neural representations associated with /aba/ versus /ada/ percepts, they do not reveal the actual structure of these neural representations. In future studies it would thus be important to combine text-based recalibration with model-based fMRI analyses^43, 44^, layer-specific fMRI^45^, and/or electrophysiological measures at the brain’s surface^46^. These approaches may reveal how recalibration effects relate to fine-grained spectro-temporal tuning properties in different areas and/or layers of the auditory cortex.

We performed a PPI analysis to investigate the relation between the brain regions active during the audiovisual exposure blocks and the superior temporal regions that subsequently entail the perceptual /aba/-/ada/ shift. This analysis showed that the correlation (functional connectivity) between bilateral IPL regions and a seed region in left STG increased during the exposure blocks compared to the baseline. This correlation may reflect the interaction between IPL and STG regions, mediating for example, the audiovisual and/or higher-order perceptual mechanisms which finally lead to the text-based recalibration effects observed during the post trials. However, since the PPI relies on correlations between hemodynamic response time-courses, our results are not conclusive on the directionality or causal nature of the underlying interactions. The involvement of the IPL would be compatible with its recruitment during experimental tasks involving the integration of spoken and written language^9, 14, 16, 18^ or cross-modal binding of familiar audio-visual mappings^32, 47^. Furthermore, functional connectivity analysis has shown bilateral IPL involvement in recalibration through lip-read speech^32^. Beyond a specific role in audiovisual binding, the IPL has also been associated with more general perceptual organization mechanisms used for the disambiguation of speech signals^48, 49^.

Although at a more lenient statistical threshold our PPI maps suggested the involvement of a larger network of brain regions including also inferior frontal and occipito-temporal regions similar to those previously observed for recalibration with lip-read speech^32^, overall our findings indicate a more confined brain network for text-based recalibration. Enhanced neural effects for lip-read speech are consistent with generally stronger behavioural recalibration effects with lip-read videos as compared to visual text^21, 25^ and are expected for naturally evolved versus acquired mechanisms for cross-modal integration^50, 51^. To gain a more detailed understanding of audiovisual networks underlying recalibration through written text versus lip-read speech, future studies could be designed to include both types of stimuli in the same participants.

In conclusion, the present study demonstrates that culturally acquired associations between written and spoken language recalibrate auditory cortical representations of speech in experienced readers. This short-term audiovisual learning involved regions in the bilateral inferior parietal lobe. Our text-based recalibration paradigm provides a novel methodological approach that uniquely enables the investigation of behavioural and neural signatures of both reading induced changes in speech perception and the audiovisual network establishing these changes. When applied in individuals with varying reading skills, including dyslexic, typical and excellent readers of different ages, this approach may reveal relevant aspects of audiovisual plasticity in the brain’s developing reading circuitry.

## Methods

### Participants

Eighteen healthy Dutch speaking adults gave their written informed consent and participated in the fMRI study. Fifteen adults were included in the analysis (mean ± SD age: 25 ± 3.1 years; 9 females; 13 right-handed). Data of 3 participants were discarded: 2 participants moved too much during functional (>4 mm) and/or anatomical measurements, and 1 participant reported perceiving /aba/ for all the stimuli during the fMRI experiment. Participants of the fMRI study all showed behavioural recalibration and adaptation effects during a preceding psychophysical experiment. The psychophysical experiment included 27 adults (27 ± 10 years; 17 females; 25 right-handed). Handedness was assessed by a handedness questionnaire adapted from Annett^52^. None of the participants had a history of neurological abnormalities and all reported normal hearing. The experimental procedures were approved by the ethics committee of the Faculty of Psychology and Neuroscience at Maastricht University, and were performed in accordance with the approved guidelines and the Declaration of Helsinki. Informed consent was obtained from each participant before conducting the experiments.

### Stimuli

Speech stimuli were based on recordings of a male Dutch speaker pronouncing the syllables /aba/ and /ada/ (see also ref. 21). The speech stimuli had a duration of 640 ms, with 240 ms stop closure, and were synthesized into a nine-token /aba/–/ada/ continuum (i.e. A1-A9) by changing the second formant (F2) in eight steps of 39 Mel using PRAAT software^53^. From this nine-token continuum, we used the three middle tokens (A4, A5 and A6; referred to as /a?a/−1, /a?a/, and /a?a/+1 respectively) for the recalibration experiments (psychophysical experiment and fMRI). During the psychophysical adaptation experiment preceding the fMRI study, we additionally used the most outer tokens (A1 and A9) corresponding to the clear /aba/ and /ada/ stimuli, respectively. Visual stimuli consisted of the written syllables ‘aba’ and ‘ada’ presented at the centre of the screen in white ‘Times New Roman’ font (font size 40) on a black background.

### Experimental design and procedure

In the fMRI study, we employed the text-based recalibration paradigm (Fig. 1) while measuring participant’s brain activity. Exposure blocks consisted of 8 trials involving the simultaneous presentation of text (‘aba’ or ‘ada’) and the ambiguous speech sound /a?a/. Audiovisual text and sound pairs were presented simultaneously (relative SOA = 0) and auditory stimuli had a duration of 640 ms, while text was presented for 1000 ms. The audiovisual exposure trials were presented with an inter-trial interval of 2 s (corresponding to 1 TR). During 6 subsequent auditory post-test trials, the most ambiguous /a?a/ sound as well as its two neighbouring sounds /a?a/−1 and /a?a/ + 1, were each presented twice in random presentation order. The post-test trials were presented in a jittered, slow event-related fashion with an average inter-trial interval of 14 s (7 TR, jitter 6–8 TR). The last audiovisual exposure trial and the first auditory post-test trial were separated by the same jittered inter-trial interval (average 14 s or 7 TR, jitter 6–8 TR), in order to also disentangle their respective brain responses. During these post-test trials, participants were asked to make forced-choice /aba/–/ada/ judgments by pressing a response button with the right index or middle finger, respectively, once the fixation cross turned green (1 s after sound onset). In total 12 ‘aba’ and 12 ‘ada’ exposure blocks were presented, each followed by 6 post-test trials, corresponding to a total of 72 post-test trials for each type of exposure block. The preceding psychophysical experiment included both recalibration and adaptation exposure blocks, which were identical in all respects, except that in the case of adaptation exposure blocks, clear /aba/ and /ada/ speech stimuli were presented together with ‘aba’ and ‘ada’ text^21, 25^. The timing of experimental trials was identical to the one used in the scanner, except that post-test trials had an average inter-trial interval of 5 s (jitter 4–6 s). The psychophysical experiment included 16 recalibration and 16 adaptation blocks, each corresponding to a total of 96 post-test trials.

### fMRI measurements

Brain Imaging was performed with a Siemens Prisma 3T MRI scanner (Siemens Medical Systems, Erlangen, Germany) using a 64-channel head–neck coil. Three 16 minute functional runs were collected (2 mm × 2 mm × 2 mm) using a multiband 3, parallel imaging (Grappa 2) echoplanar-imaging (EPI) sequence (repetition time [TR] = 2000 ms, acquisition time [TA] = 1300 ms, field of view [FOV] = 192 mm × 192 mm, echo time [TE] = 29 ms). Each volume consisted of 63 slices (no gap), covering the whole brain, except the most superior tip of the posterior parietal cortex in some participants. Speech stimuli were presented binaurally at a comfortable listening level via MR compatible headphones (Sensimetrics, model S14, www.sens.com), in the 700-ms silent gap between consecutive volume acquisitions (Fig. 1). During audiovisual exposure blocks, text and speech stimuli were presented once every TR (8 trials per block). Each of the three functional runs contained 4 ‘aba’ and 4 ‘ada’ exposure blocks presented in random order. The auditory post-test trials (6 trials per block) were presented according to a slow event-related design with an average inter-trial-interval of 14 s (range 12 to 16 s). We additionally collected a high-resolution structural scan (1 mm × 1 mm × 1 mm) using a T1-weighted three-dimensional MPRAGE sequence ([TR] = 2250 ms, [TE] = 2.21 ms, 192 sagittal slices).

### fMRI pre-processing

Functional MRI data were subjected to conventional pre-processing in BrainVoyager QX 2.8 (Brain Innovation). Slice scan-time correction was performed with respect to the first slice of each volume using sinc interpolation and data were high-pass temporal filtered to remove nonlinear drifts of five or less cycles per time course. Three-dimensional motion correction was performed by spatial alignment of all volumes of a subject to the first volume of the second functional run of each session by rigid body transformations. Preprocessed functional data were then co-registered to each individual subject’s structural images and both anatomical and functional data were normalized to Talairach space^54^. For all included participants, estimated head movements were within one voxel (2 mm) in any direction. Based on the anatomical scans, individual cortical surfaces were reconstructed from grey–white matter segmentations. An anatomically aligned group-average cortical surface representation was obtained per hemisphere by aligning all 15 individual cortical surfaces using a moving target-group average approach based on curvature information (cortex-based alignment^54^). In order to map fMRI signal time courses from volume space to surface space, values located between the grey/white matter boundary and up to 3 mm into grey matter towards the pial surface were sampled with trilinear interpolation and averaged, resulting in a single value for each vertex of a cortex mesh.

### Whole brain univariate fMRI analysis

Random effects (RFX) general linear model (GLM) analyses were performed on time course data sampled on individual cortical surface meshes, aligned to the cortical group surface mesh using cortex-based alignment. Our first-level GLM model included one predictor for each audiovisual exposure block type and single-trial predictors for each auditory post-test trial (convolved with a double gamma hemodynamic response function with standard values). The overall pattern of fMRI responses during the audiovisual exposure blocks and auditory post-test trials was assessed by calculating functional contrast maps (t-statistics; exposure blocks > baseline; post-test trials > baseline). Functional contrast maps were corrected for multiple comparisons by applying a surface-based cluster-size threshold correction. For the latter, an initial vertex-level threshold of p_vertex_ = 0.001 was selected and maps were submitted to a whole-brain correction criterion (p_corr_ < 0.05) based on Monte Carlo simulations (5000 iterations) which also accounted for the estimated map’s spatial smoothness^54, 55^.

### Multivariate fMRI analysis of auditory post-test trials

Multivoxel patterns of speech sound-evoked fMRI responses during the post-trials were analysed by applying a machine learning algorithm (support vector machine, SVM^56^) with an iterative multivariate voxel selection algorithm, Recursive Feature Elimination (RFE^57^) within an anatomically defined broad mask encompassing the superior temporal cortex (STC) (see blue outlines in Fig. 4b). The same anatomical STC mask was projected and applied across subjects and included all temporal cortical activity to the speech sounds. The left and right hemisphere ROIs were constructed such that they contained equal numbers of vertices. Although it would be interesting to also investigate the involvement of sensorimotor mechanisms in shifting the perceived phoneme category, our anatomical mask excluded sensorimotor and motor regions because activity in these regions was associated with participants’ button presses indicating /aba/ versus /ada/ perceptions.

#### Classification procedure

To assess the capacity of the fMRI decoding algorithm to discriminate superior temporal cortical activity associated with /aba/ versus /ada/ perception, preprocessed functional time series were divided into individual “trials” with respect to the ambiguous post-test sound and labelled according to participant’s perceptual responses (/aba/ or /ada/ response). Voxel-trial features for classification were calculated using beta estimates of the fitted double-gamma hemodynamic response with respect to sound onset using a temporal adjustment of the positive-time-to-peak independently per voxel (between 3.2 and 4.2 s). Because the number of /aba/ versus /ada/ perceptions was not always balanced at the single subject level (mean ratio of /aba/ versus /ada/ perceptions = 1.50, SD = 1.57), we created 10 datasets with evenly represented classes by randomly picking (with replacements) the number of trials from the least represented class from the most represented class. For each balanced dataset, training and testing sets were created using 4 independent folds (‘k-fold’ method), resulting in a total of 40 cross-validation folds. Voxel-trial features included in each training set were normalized (z-score) across trials and the resulting mean and standard deviation were applied to the respective testing set. Feature selection and multivariate classification were performed iteratively using recursive feature elimination (RFE) with 10 feature selection levels and an elimination ratio of 30%. RFE involved further splitting of each cross-validation fold into 50 splits by randomly picking 90% of the training trials. For each RFE selection level, 5 splits were used and their classification outcomes were averaged. Within each selection level, the cortical weight maps were smoothed using a Gaussian filter (SD = 5 mm), normed to positive values, and ranked for subsequent feature elimination^57^. Classification was performed using linear support vector machine classifiers (SVM^56^) as implemented in the Bioinformatics Matlab toolbox, using the sequential minimal optimization method.

#### Statistical Testing

To test whether classification values were significantly above chance, we performed the exact same multivoxel pattern analysis as described above with randomly shuffled condition labels within the training set per subject (number of permutations = 200). At the group level, statistical significance was assessed by comparing the single-subject accuracies of perceptual label (/aba/ vs. /ada/) classification with the average permutation accuracy of the respective subjects using a non-parametric Wilcoxon test (two-tailed).

#### Mapping of Informative Regions

We constructed discriminative maps of STC locations that contributed most to classification of the perceptual labels. The RFE level in which each feature was eliminated for classification was used to create a map of relative discriminative contribution for each cross-validation fold and subject. These cortical maps, averaged across folds, were subsequently projected on the group-averaged cortex-based aligned cortical surface mesh. Finally, inter-individual consistency maps were created by indicating the number of subjects for which each vertex was among the 20% (~2000) most discriminative features of the ROIs.

### Functional connectivity analysis of audiovisual exposure block activity

To investigate the functional dynamics of audiovisual brain activity during the exposure blocks we performed a psychophysiological interaction analysis (PPI^31^) and modelled task-dependent cortico-cortical connectivity with a seed region in the left STC. The seed region corresponded to the left STC region that most consistently contributed to the decoding of /aba/ vs. /ada/ perceptions of the auditory post-test trials. The PPI analysis was performed by adding two predictors to our first-level GLM model, a predictor for the activity time course in the seed region (z-scored) and one for the interaction between the task (audiovisual exposure blocks, z-scored) and the time course of the seed (PPI). The PPI predictor served to identify regions that showed increased functional connectivity with the left STC region during the audiovisual exposure blocks relative to baseline. Note that because the PPI model includes predictors accounting for the audiovisual exposure blocks, the PPI predictor explains additional variance to that explained by the voxel responses to audiovisual exposure blocks. Functional group maps were created by running this analysis as an RFX GLM on the cortex-based aligned data. Statistical significance was assessed using vertex-wise t-statistics and corrected for multiple comparisons by applying the surface-based cluster-size threshold correction described above, with an initial vertex-level threshold of p_vertex_ = 0.005 or 0.001 and a whole-brain correction criterion of p_corr_ < 0.05.

### Data Availability

The datasets generated and analysed during the current study are available from the corresponding author on reasonable request.
